# Supplementary material for: Adolescents’ reports of chaos within the family home environment: Investigating associations with lifestyle behaviours and obesity
Source: PLoS One. 2023 Jan 26;18(1):e0280737. doi: 10.1371/journal.pone.0280737 (PMC9879426; doi:10.1371/journal.pone.0280737)
Supplement: S1 Table — (DOCX) [file pone.0280737.s001.docx]

# **Online Supplemental Data**

**Title:** Adolescents’ reports of chaos within the family home environment: investigating associations with lifestyle behaviours and obesity

**Authors :** Andraea Van Hulst,^1*^ Sujani Jayanetti,^1^ Ana Maria Sanson-Rosas,^1^ Marie-Josée Harbec^2^, Lisa Kakinami,^3,4^ Tracie A Barnett,^5,6^ Mélanie Henderson,^6,7,8^

**S1 Table. Item-score correlations for the 15 items included in the Confusion, Hubbub, and Order Scale^1^ (CHAOS), QUALITY cohort study (n=377)**

| Item | Item-score correlation |
| --- | --- |
| 1. There is very little commotion in our home. | 0.41** |
| 2. We can usually find things when we need them. | 0.23** |
| 3. We almost always seem to be rushed. | 0.42** |
| 4. We are usually able to stay on top of things. | 0.39** |
| 5. No matter how hard we try, we always seem to be running late. | 0.20** |
| 6. It’s a real zoo in our home. | 0.54** |
| 7. At home we can talk to each other without being interrupted. | 0.45** |
| 8. There is often a fuss going on at our home. | 0.54** |
| 9. No matter what our family plans, it usually doesn’t seem to work out. | 0.40** |
| 10. You can’t hear yourself think in our home. | 0.44** |
| 11. I often get drawn into other people’s arguments at home. | 0.46** |
| 12. Our home is a good place to relax. | 0.56** |
| 13. The telephone takes up a lot of our time at home. | 0.33** |
| 14. The atmosphere in our home is calm. | 0.57** |
| 15. First thing in the day, we have a regular routine at home. | 0.31** |

** Correlation is statistically significant at the 0.01 level (two-tailed).

Item-total score correlations revealed weak to moderate correlations between each item of the CHAOS questionnaire and the total scale. In this sample, Cronbach’s alpha for the item-score correlation was 0.64. Item 2 (we can usually find things when we need them) and item 5 (no matter how hard we try, we always seem to be running late) displayed the weakest correlation at 0.23 and 0.20 respectively. However, removing items 2 and 5 did not improve Cronbach’s alpha.

^1.^ Matheny AP, Wachs TD, Ludwig JL, Phillips K. Bringing order out of chaos: Psychometric characteristics of the confusion, hubbub, and order scale. Journal of Applied Developmental Psychology. 1995;16(3):429-44.
