# Supplementary material for: Adolescents’ reports of chaos within the family home environment: Investigating associations with lifestyle behaviours and obesity
Source: PLoS One. 2023 Jan 26;18(1):e0280737. doi: 10.1371/journal.pone.0280737 (PMC9879426; doi:10.1371/journal.pone.0280737)
Supplement: Attachment — Submitted filename: 2022-11-10 CHAOS in QUALITY_Response to editors.docx [file pone.0280737.s002.docx]

Rebuttal Letter to PLOS ONE

Manuscript Number: PONE-D-22-15790

Title: Adolescents’ reports of chaos within the family home environment: investigating associations with lifestyle behaviours and obesity

Dear Dr. Linglin Xie,

In response to the comments from the editorial team to the above-mentioned manuscript, we are pleased to provide you with a revised data accessibility statement for our manuscript.

We hope that these revisions will be found suitable for our manuscript to be considered for publication in PLOS ONE.

**Editorial comments**

**Comment 1.** Thank you for updating your Data Availability statement. Can you clarify on this statement: "When will data be available (start and end dates)?
Beginning 3 months and ending 6 months following article publication"

Please note that PLOS journals require authors to make all minimal data necessary to replicate their study’s findings publicly available without restriction at the time of publication, with specific legal or ethical exceptions: <https://journals.plos.org/plosone/s/data-availability>.

It would not be acceptable for data access to be discontinued at a specific point after publication.

If there are no legal or ethical restrictions on the public sharing of your data, please upload your data either as a Supporting Information file or to a stable, public repository. PLOS holds a list of recommended repositories here: <https://journals.plos.org/plosone/s/recommended-repositories>

If there are in fact legal or ethical restrictions, please provide the name of the ethics committee or Institutional Review Board that is imposing sharing restrictions on your minimal data set, the reason for restriction, and a non-author point of contact that is able to receive queries regarding data access.

**Response:** Please find below an updated Data availability statement that we hope will be found acceptable for the journal. As mentioned earlier, for ethical reasons, data from study participants cannot be shared openly as they include potential identifying participant information. Moreover, participants have not provided consent for data to be deposited in a public repository. Our revised statement was validated with the Research Ethics Board that provided initial approval for the QUALITY Cohort study, that is the Research Ethics Board of the CHU Sainte-Justine Hospital presided by Me Geneviève Cardinal ([genevieve.cardinal.hsj@ssss.gouv.qc.ca](mailto:genevieve.cardinal.hsj@ssss.gouv.qc.ca)). We now also offer a non-author point of contact who can receive queries regarding data access.

Data accessibility statement:

| Will individual participant data be available (including data dictionaries)? | Data will not be shared in a public repository but will be made available upon reasonable requests as per the conditions outlined below. |
| --- | --- |
| What data in particular will be shared? | Individual participant data that underlie the results reported in this article, after de-identification (text, tables, figures, and appendices). |
| What other documents will be available? | Study protocol, analytic code |
| When will data be available (start and end dates)? | Beginning 3 months after publication of the article and until the research team is able to maintain the data. |
| With whom? | Investigators whose proposed use of the data has been approved by the executive committee of the QUALITY cohort study. |
| For what types of analyses? | To replicate findings from the current secondary analysis project. |
| By what mechanism will data be made available? | To gain access, data requestors will need to sign a data access agreement. Requests should be directed to the QUALITY Cohort Study Principal Investigator Dr. Mélanie Henderson at [melanie.henderson.hsj@gmail.com](mailto:melanie.henderson.hsj@gmail.com), or to Catherine Pelletier (Study Coordinator) at [catherine.pelletier.hsj@ssss.gouv.qc.ca](mailto:catherine.pelletier.hsj@ssss.gouv.qc.ca) |

We hope that the modifications brought to the manuscript will meet the editorial team and reviewers’ satisfaction and qualify for publication.

Sincerely,

Andraea Van Hulst, PhD, RN

Assistant Professor

McGill University Ingram School of Nursing

680 Sherbrooke West, Office 1833

Montreal, QC, Canada H3A 2M7
